# The impact of adherence on colorectal cancer screening cost-effectiveness: A modeling study

**DOI:** 10.1371/journal.pmed.1004807

**Published:** 2025-11-26

**Authors:** Jiaxin Xie, Xuesi Dong, Zilin Luo, Chenran Wang, Yadi Zheng, Xiaolu Chen, Zeming Guo, Xiaoyue Shi, Fei Wang, Wei Cao, Yongjie Xu, Le Wang, Weimiao Wu, Dong Hang, Lingbin Du, Ni Li

**Affiliations:** 1 Office of Cancer Screening, National Cancer Center/National Clinical Research Center for Cancer/Cancer Hospital, Chinese Academy of Medical Sciences and Peking Union Medical College, Beijing, China; 2 Chinese Academy of Medical Sciences Key Laboratory for National Cancer Big Data Analysis and Implement, Chinese Academy of Medical Sciences and Peking Union Medical College, Beijing, China; 3 Department of Cancer Prevention, Zhejiang Cancer Hospital, Hangzhou Institute of Medicine (HIM), Chinese Academy of Sciences, Hangzhou, Zhejiang, China; 4 Department of Epidemiology, Jiangsu Key Lab of Cancer Biomarkers, Prevention and Treatment, Collaborative Innovation Center for Cancer Personalized Medicine, School of Public Health, Nanjing Medical University, Nanjing, China; 5 Department of Epidemiology and Biostatistics, Jiangsu Key Lab of Cancer Biomarkers, Prevention and Treatment, Collaborative Innovation Center for Cancer Personalized Medicine, School of Public Health, Nanjing Medical University, Nanjing, China; University of Calgary, CANADA

## Abstract

**Background:**

Adherence to colorectal cancer (CRC) screening remains suboptimal in many countries, reducing its cost-effectiveness. This study aimed to evaluate how multistage uptake rates influence the health benefit and cost-effectiveness of various CRC screening strategies in the Chinese population, incorporating both traditional and emerging screening methods.

**Methods and findings:**

We developed a multistate Markov model (CRC-SIM) to evaluate the impact of multistep uptake on CRC screening. A hypothetical cohort of 100,000 individuals aged 40 was simulated and followed until 79 or death. Two-step screening strategies were modeled: initial screening followed by colonoscopy after a positive result. Traditional initial screening methods include: questionnaire-based risk assessment, fecal immunochemical test (FIT), and questionnaire combined with FIT; Non-invasive biomarker-based initial strategies include a hypothetical test meeting the minimum standards of China National Medical Products Administration (*NMPA*_*min*_), multitarget stool DNA (mt-sDNA) test, and blood-based strategies. All strategies were modeled as one-time screenings, with outcomes projected for CRC cases, deaths, quality-adjusted life years (QALYs), and lifetime costs. Incremental cost-effectiveness ratios (ICERs) were calculated, and a cost-effectiveness heatmap was conducted to assess the impact of multistep uptake (modeled in 10% steps) on economic outcomes. All strategies reduced CRC cases, deaths and increased QALYs compared to no screening, with biomarker-based strategies outperforming the traditional methods at the same uptake level (e.g., questionnaire combined with FIT prevented 224 (95% confidence interval (CI) [157, 292]) CRC cases and 151 (95% CI [109, 195]) deaths, whereas NMPA_*min*_ prevented 312 (95% CI [257, 360]) cases and 210 (95% CI [175, 241]) deaths at 100% uptake). The cost-effectiveness heatmap indicated that each 10% increase in initial and follow-up colonoscopy uptake improved ICERs in a non-linear pattern. The questionnaire combined with FIT was the most cost-effective strategy (ICER = $2,413 per QALY gained). Non-invasive biomarker-based tests were not cost-effective compared with the combined questionnaire and FIT strategy under current assumptions of test costs and identical uptake rate. Threshold analysis showed that non-invasive biomarker-based screening would become cost-effective if test costs fell below $131.7 or colonoscopy uptake increased to at least 70% for *NMPA*_*min*_ and 50% for blood-based tests and mt-sDNA. Limitations include the assumption of a one-time screening scenario; future iterations of the model and merging evidence in repeated screening will address these limitations.

**Conclusion:**

Improving screening participation could enhance health benefits and cost-efficiency in CRC screening. Questionnaire-based risk assessment combined with FIT was a cost-effective strategy in China, whereas non-invasive biomarker-based methods require cost reduction and higher uptake to justify adoption. These findings provide evidence for policymakers to optimize CRC screening programs.

## Author summary

**Why was this study done?:**

- The burden of colorectal cancer (CRC) in China has been growing in recent years.
- Suboptimal adherence rates compromise the health benefit and the cost-effectiveness of CRC screening.
- The quantitative relationship between multistage adherence in CRC screening and its long-term health and cost-effectiveness benefit remains insufficiently evaluated.

**What did the researchers do and find?:**

- Leveraging a well-validated multistate Markov model, we quantified the impact of multistage adherence on the long-term health and economic benefit, incorporating both traditional and emerging biomarker-based initial screening modalities.
- Our analysis demonstrated that enhancing adherence rate at both the initial risk assessment and follow-up colonoscopy stages yields substantial health benefits and cost-effectiveness gains.
- Questionnaire-based risk assessment combined with fecal immunochemical test was a cost-effective strategy in China, while non-invasive biomarker-based tests were not cost-effective unless costs decreased or follow-up adherence improved.

**What do these findings mean?:**

- Strengthening adherence to CRC screening across multiple stages is critical to maximizing public health and economic benefits.
- CRC screening strategies should be tailored to the adherence characteristics of target populations.
- A key limitation is the assumption of a one-time CRC screening scenario, without considering repeated screening for some strategies, making our study more relevant to settings with limited resources and suboptimal follow-up adherence.

## Abstract 3

Jiaxin Xie, Xuesi Dong, Zilin Luo, and team use a multistate Markov model to quantify the long-term health and economic benefits of adhering to colorectal cancer screening, incorporating both traditional and biomarker-based initial screening methods.

## Introduction

Colorectal cancer (CRC) is the third most common cancer worldwide and the second leading cause of cancer-related mortality, with an estimated 1.93 million new cases and 0.90 million deaths in 2022 [1]. China experienced a substantial burden of CRC, accounting for around 30% of the global burden [2]. According to data from the National Cancer Center of China, over 517,100 new CRC cases and 240,000 deaths occurred in 2022 [2]. Although screening is effective in reducing CRC mortality [3], optimizing its implementation is essential to improving the cost-effectiveness of population-based screening programs.

Colonoscopy is the gold standard for CRC screening; however, its widespread use as a primary screening modality could be limited by resource constrained, high complications, and low adherence rates [4–6]. Regions with constrained resources consequently adopt a two-step screening process: an initial phase entails low-invasive techniques to identify high-risk populations from healthy individuals, followed by the subsequent colonoscopy [3,7]. Low-invasive tools commonly used in the initial phase include risk assessment questionnaires and fecal occult blood tests. More recently, blood- and stool-based biomarkers have emerged as promising alternatives for high-risk identification, offering potentially higher accuracy and improved uptake [8,9].

The uptake rates among initial screening and follow-up colonoscopy are important to increase screening efficacy and cost-effectiveness. Currently, the uptake rates of screening recommendations remain suboptimal; the uptake for colonoscopy among high-risk individuals ranges from 14% to 26.5% [10–13]. Non-adherence at initial risk assessment and follow-up colonoscopy could lead to missed diagnoses, delayed treatments, and reduced cost-effectiveness [14]. Moreover, an increase in uptake rate typically needs higher expenditures. Policymakers are constantly faced with the challenge of health benefits and fiscal expenditure trade-offs at varying uptake rates [15]. The expected long-term effectiveness and cost-effectiveness of screening under multilevel, especially under suboptimal adherence, and multistage adherence scenarios remain underexplored [13,16,17].

Simulation modeling studies can provide valuable guidance for optimizing CRC screening and informing guideline updates [18]. By synthesizing data on epidemiology, disease progression, intervention, and healthcare costs, these models could evaluate the simulated target scenarios and quantify the long-term efficacy and cost-effectiveness impact of various uptake rates [19–21]. However, most studies rely on simplified assumptions of optimal uptake rates (100%) or neglect the comprehensive impact of multiple-stage uptake rates across screening processes [22,23].

To address these gaps, we construct a multistate Markov model to simulate the long-term health benefits and cost-effectiveness impact of the uptake rate on CRC screening. Our findings could help policymakers optimize resource allocation and strengthen quality management in screening programs.

## Materials and methods

### Markov model overview

**Fig 1:**
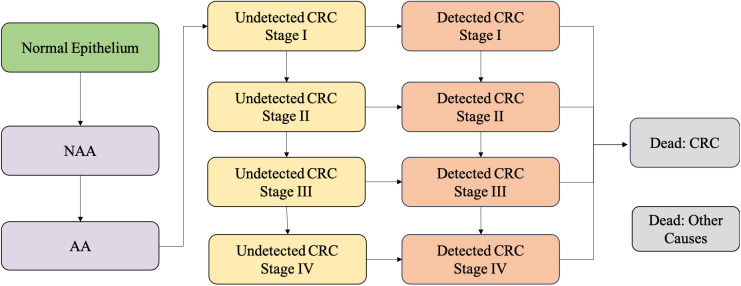
Schematic of the colorectal cancer screening simulation (CRC-SIM) model. Abbreviations: NAA, non-advanced Adenoma; AA, advanced adenoma; CRC, colorectal cancer.

We developed and validated a Markov-based colorectal cancer screening simulation (CRC-SIM) model to simulate the natural history, screening, and clinical management of CRC adapted to the Chinese population. The Markov structure facilitates dynamic health states transitions and long-term evaluation of various “what-if” scenarios for medical intervention [24]. CRC-SIM consists of nine mutually health states: normal epithelium, non-advanced adenoma, advanced adenoma (10 mm, with villous components, or high-grade intraepithelial neoplasia), CRC stages I–IV, CRC-related death, and death from other causes (Fig 1) [25,26]. A population cohort with 100,000 hypothetical individuals was simulated, with all individuals entering the model at the age of 40 years, in alignment with the starting age of national programs under China’s Basic Public Health Service. The individuals were followed up until age 79 (the average life expectancy of the Chinese population [27]) or death, whichever occurred first [13,28]. At the start of the simulation, certain proportions of no neoplasm, non-advanced adenoma, advanced adenoma, and CRC (stage I–IV) are assigned to the hypothetical population. Each time cycle of the model (1 year), individuals have the probability of transitioning between health states. If an individual were diagnosed, the disease stops progressing, and this individual should follow a disease pathway. Screening and removal of adenomas can prevent the occurrence of CRC, while preclinical CRC will be regarded as screening-detected CRC. Individuals with a positive diagnostic colonoscopy enter a surveillance scheme. Subjects will then continue to have the probability to progress to the next states, as those without findings at screening. The detailed components and assumptions were detailed in the S1 Text.

### Model parameters

The model parameters included transition probabilities, health utilities, and costs. Transitional probabilities for the natural history model were derived from systematic literature reviews of Chinese studies [29–32]. Data from large-scale clinical studies in China were utilized to estimate survival outcomes and adverse event rates after treatment (Table A in S1 Text) [33]. The age-specific all-cause mortality was derived from the China Statistics Yearbook. Quality-adjusted life years (QALYs) were calculated as life expectancy weighted by utility [34]. Utility scores for each health state, ranging from 0 (death) to 1 (normal epithelium), were obtained from a hospital-based cross-sectional study on 300 newly diagnosed colorectal neoplasm patients using EQ-5D-5L in China (Table 1) [35,36].

**Table 1:** Health utility for different health status.

Parameters	Base case value	Source
No colorectal lesion and False positive	1.000	[33,35]
NAA	0.955	
AA	0.955	
CRC		
CRC stage I	0.768	
CRC stage II	0.656	
CRC stage III	0.562	
CRC stage IV	0.495	
Death	0	

*Abbreviations:* CRC, colorectal cancer; AA, advanced adenoma; NAA, non-advanced adenoma.

From a societal perspective, cost components include (1) direct medical expenses from clinical diagnoses and treatments; (2) travel expenses; (3) productivity losses; and (4) costs specific to screening procedures [33]. Cost estimates were sourced from population-based CRC screening programs in China, as well as a multicenter, cross-sectional survey in 37 tertiary hospitals in 13 provinces across China (Table B in S1 Text) [13,33]. All costs were adjusted based on the consumer price index in China and subsequently converted into US dollars (1 USD = 7 RMB).

To accurately simulate expected CRC epidemiology in China, parameters were calibrated to targeted data from the Global Burden of Disease database (https://www.healthdata.org/) and China’s National Cancer Center registry, including age-specific CRC incidence, mortality, and stage distribution [37,38]. Bound optimization BY quadratic approximation, a derivative-free optimization algorithm, was used to search for an optimal parameter solution by minimizing the weighted sum of squared differences between the observed and simulated data [39,40]. Model outputs were consistent with the targeted observation (Figs A and B in S1 Text).

### Screening strategies and characteristics

Based on current CRC screening practices, six initial screening modalities were considered before the follow-up colonoscopy for high-risk individuals, including conventional approaches: (1) questionnaire-based risk factors assessment, (2) fecal immunochemical tests (FITs), and (3) questionnaire-based risk factors assessment combined with FIT outcomes. Additionally, non-invasive biomarker-based strategies were evaluated: (4) a hypothetical non-invasive test that meets the minimum standards established by China National Medical Products Administration (NMPA), (5) a multitarget stool DNA (mt-sDNA) test, and (6) blood-based strategies [3,7,41]. Individuals were scheduled to undergo one-time screening at age 40 across all screening modalities, aligning with current CRC screening practice in China (Table C in S1 Text). We further assumed that non-adherent individuals did not receive further screening. The sensitivity and specificity for initial screening were derived from published literature or systematic reviews on Chinese populations (Table 2) [42–45]. Baseline performance characteristics for non-invasive tests were derived from the decision criteria outlined by NMPA, with a sensitivity of 85% for CRC, 50% for advanced adenoma, and a CRC specificity of 90%. Besides, we comprehensively collected performance data for mt-sDNA and blood-based tests approved by NMPA [46–51]. The performance data were updated based on a meta-analysis of approved products (Table D in S1 Text). Follow-up colonoscopy parameters were sourced from a recent meta-analysis [4].

**Table 2:** Screening test characteristics used in the analysis.

Strategy	Sensitivity				Specificity	Source
	NAA	AA	Preclinical CRC & CRC (Stage I-II)	Preclinical CRC & CRC (Stage III- IV)		
Colonoscopy	85%	95%	95%		86%	[4]
FIT	8.7%	20.3%	44.6%	78.9%	87.4%	[45]
RF	13%	14%	10%		87.62%	[43]
RF-FIT	9.4%	33%	74.2%	93.1%	79.3%	[45]
Biomarker-based test						
*NMPA*_*min*_	10%^a^	50%	85%		90%	NMPA
Blood-based test	13%	61%	86%		90 %	Meta-analysis [50,51]
mt-sDNA test	14%	57%	91%		91%	Meta-analysis [46–49]

Abbreviations: RF, risk factors questionnaire; FIT, fecal immunochemical tests; CRC, colorectal cancer; RF-FIT, risk factors questionnaire combined with FIT; AA, advanced adenoma; NAA, non-advanced adenoma; NMPA, China National Medical Products Administration. *NMPA*_*min*_: a hypothetical novel biomarker-based test that complies with the minimum standards established by the China National Medical Products Administration.

^a^Adenomas are only detected by chance, with sensitivity set to the positivity rate in people without adenomas or cancer (1 − specificity).

### Cost‑effectiveness analysis

We projected CRC cases, death cases, QALYs, lifetime costs, and the incremental cost-effectiveness ratio (ICER) for each screening strategy. QALYs were calculated as life years weighted by utility. Strategies that provided fewer QALYs at a higher cost than any alternative were considered dominated; Strategies yielding more QALYs at a lower cost dominate. ICERs were calculated by dividing the incremental costs by the incremental QALYs for each strategy, compared either to no screening or to the next most effective alternative (i.e., the most cost-effective option among the remaining lower-cost strategies). A screening strategy would consider to be considered cost-effective if the ICER was less than the predefined willingness-to-pay (WTP) threshold of the 2022 per capita gross domestic product in China per QALY gained (US$12,246) [52]. Cost-effectiveness frontier was performed to identify the optimal screening strategies among the evaluated strategies. All health and cost outcomes were calculated and discounted at 5% according to the China Guidelines for Pharmacoeconomic Evaluations and with the application of half-cycle correction [34].

### Uptake rate to initial screening and follow-up colonoscopy

Analyses considered 2 uptake rate scenarios: (1) In the base case analysis, an idealized implementation scenario were simulated as a reference, assuming 100% initial uptake and 100% followed-up colonoscopy rate after a positive noninvasive test; (2) Additionally, a more realistic spectrum of random uptake rates was assumed, indicating that the entire population has a probability *p* (ranging from 0% to 100%) of adherence to each part of the screening pathway (initial screening test *and* follow-up colonoscopy) independent of previous behavior, to assess the impact on the effective and cost-effectiveness of screening programs [13,42,53].

Finally, we generated a two-dimensional uptake health benefit and cost-effectiveness “heatmap” to visualize the joint effects of initial screening and follow-up colonoscopy uptake rates on prevented CRC death cases and ICER, with color gradients indicating corresponding values. This tool can assist test developers and policymakers in identifying the expected adherence thresholds for achieving cost-effective outcomes. Accordingly, cost-effectiveness non-inferiority was defined as achieving at least the same cost-effectiveness benefit as the predefined WTP threshold relative to no screening, at specific adherence levels. Cost-effectiveness noninferiority is the same as non-dominance.

### Sensitivity analysis

Extensive deterministic sensitivity analysis was performed to assess the robustness of the model and identify the most sensitive parameters, including screening performance (sensitivity, specificity, and complications rate), costs (screening, examinations, treatments, follow-up), and health utility. Tornado diagrams identified the parameters with the strongest impact on the ICER in one-way sensitivity analyses. Probabilistic sensitivity analysis was performed using 10,000 Monte Carlo simulations and was used to calculate the 95% confidence intervals (CIs).

This study is reported as per the Strengthening the Consolidated Health Economic Evaluation Reporting Standards 2022 (CHEERS 2022) Statement (Table E in S1 Text) [54]. The model was conducted in TreeAge 2022 R2.1 (TreeAge Software, Williamstown, MA), and all statistical analyses were performed in R (Version 4.0.3, R Foundation for Statistical Computing, Vienna, Austria).

## Results

### Health benefit and cost-effectiveness outcomes with uptake rates of 100%

All screening modalities were effective in reducing CRC cases and deaths and yielded QALY gains, with non-invasive biomarker-based strategies outperforming conventional approaches. Conventional methods, include questionnaire-based assessment, FIT, and combined RF-FIT strategies, prevented 154 (95% CI [124, 179]), 157 (95% CI [114, 199]), and 224 (95% CI [157, 292]) CRC cases, and 90 (95% CI [74, 104]), 102 (95% CI [76, 130]), and 151 (95% CI: [109, 195]) deaths per 100,000 persons compared with no screening. Non-invasive biomarker-based strategies, including the minimum standards of China National Medical Products Administration ([*NMPA_min_*], blood-based, and mt-sDNA tests, prevented 312 (95% CI [257, 360]), 383 (95% CI [259, 493]), and 384 (95% CI [312, 441]) CRC cases, and 210 (95% CI [175, 241]), 253 (95% CI [174, 324]), and 255 (95% CI [208, 290]) deaths per 100,000 persons, respectively, compared with no screening (Table 3).

**Table 3:** Clinical and cost-effectiveness of screening strategies, in a hypothetical cohort of 100,000 persons.

Variable	No screen	RF	FIT	RF-FIT	NMPA_min_	Blood-based	mt-sDNA
**Outcome**							
CRC cases	4,076 (4,076, 4,076)	3,922 (3,897, 3,952)	3,919(3,877, 3,962)	3,852 (3,784, 3,919)	3,764 (3,716, 3,819)	3,693 (3,583, 3,817)	3,692(3,635, 3,764)
Prevented CRC case vs. no screen		154 (124, 179)	157 (114, 199)	224 (157, 292)	312 (257, 360)	383 (259, 493)	384 (312, 441)
CRC deaths	1813 (1813, 1813)	1,723(1,709, 1,739)	1,711(1,683, 1,737)	1,662 (1,618, 1,704)	1,603 (1,572, 1,638)	1,560 (1,489, 1,639)	1,558 (1,523, 1,605)
Prevented CRC deaths vs. no screen		90 (74, 104)	102 (76, 130)	151 (109, 195)	210 (175, 241)	253 (174, 324)	255 (208, 290)
QALYs/person	16.019 (15.8, 16.182)	16.028 (15.813, 16.189)	16.028 (15.812, 16.19)	16.032 (15.815, 16.195)	16.037 (15.82, 16.199)	16.040 (15.825, 16.202)	16.041 (15.825, 16.202)
Costs/person (US$)	199.410 (194.149, 204.835)	222.249 (210.465, 239.49)	218.209 (206.503, 235.509)	227.127 (214.45, 242.739)	356.775(302.475, 424.675)	353.821 (298.343, 419.959)	353.165 (299.055, 420.937)
**Cost(US$)/QALY gained versus:**							
No screen	——	dominated	1,951	2,413	dominated	dominated	6,877
RF-FIT	——	——	——	——	dominated	dominated	14,321

Values in parentheses indicate 95% confidence intervals (95% CIs). *Abbreviations:* CRC, colorectal cancer; QALY, quality-adjusted life year; ICER, incremental cost-effectiveness ratio. The following initial screening strategies were considered, including questionnaire-based risk factors assessment (RF), fecal immunochemical tests (FIT), questionnaire-based risk factors assessment combined with FIT outcomes (RF-FIT), a hypothetical non-invasive test that meets the minimum criteria set by China National Medical Products Administration (NMPA_min_), blood-based strategies, and multitarget stool DNA (mt-sDNA) test. Dominated refers to dominated strategies that provided fewer QALYs at a higher cost than any alternative.

The average lifetime cost per person was $199.410 (95% CI [194.149, 204.835]) without screening (Table 3). For questionaries, FIT, and their combinations, the projected lifetime costs are $222.249 (95% CI [210.465, 239.49]), $218.209 (95% CI [206.503, 235.509]), and $227.127 (95% CI [214.45, 242.739]), while costs increased for *NMPA*_*min*_, blood-based, and mt-sDNA strategies, reaching $356.775 (95% CI [302.475, 424.675]), $353.821 (95% CI [298.343, 419.959]), and $353.165 (95% CI [299.055, 420.937]) per person. All strategies were cost-effective versus no screening at the $12,246/QALY threshold, yet non-invasive biomarker-based strategies were suboptimal compared to questionnaire-based assessment combined with FIT (the most cost-effective strategy) (ICERs: $14,321) (Figs C and D in S1 Text).

### Impact of uptake rates on screening health benefit and cost-effectiveness

**Fig 2:**
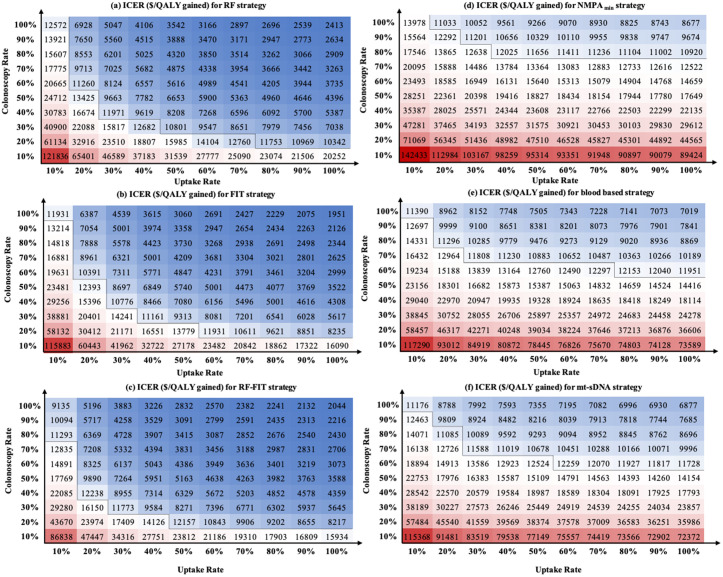
Impact of uptake rate on cost-effectiveness of various colorectal cancer screening strategies in China. The following initial screening strategies were considered, including questionnaire-based risk factors assessment (RF), FIT, questionnaire-based risk factors assessment combined with FIT outcomes (RF-FIT), a hypothetical non-invasive test that meets the minimum criteria set by China National Medical Products Administration (*NMPA*_*min*_), blood-based strategies, and mt-sDNA test. The incremental cost-effectiveness ratio (ICER, $/QALY gain) was calculated for each screening modality in comparison to no screening, accounting for the uptake rates for both initial screening and follow-up colonoscopy. The black line indicates the willingness-to-pay threshold ($12,246 per QALY gained); ICERs below this value are considered cost-effective. Red represents ICERs above the willingness-to-pay threshold, with darker shades corresponding to higher ICERs and lower cost-effectiveness; blue represents ICERs below the threshold, with darker shades corresponding to lower ICERs and higher cost-effectiveness. Abbreviations: ICER, incremental cost-effectiveness ratio; FIT, fecal immunochemical test; NMPA, China National Medical Products Administration; mt-sDNA, multitarget stool DNA.

The health benefit and cost-effectiveness heatmap was developed to quantify the effect of multistep uptake rate on CRC screening (Figs 2 and E in S1 Text). Briefly, higher uptake rates of initial screening and follow-up colonoscopy could substantially prevent more CRC deaths and improve cost-effectiveness across all screening strategies (ICER decreased). For questionnaire-based assessment combined with the FIT strategy, the ICER decreased substantially as uptake improved, ranging from $86,838 per QALY gained with 10% uptake for initial screening and 10% follow-up colonoscopy to $2,044 per QALY gained with 100% uptake for both. For non-invasive screening modalities, e.g., *NMPA*_*min*_, ICER decreased from $142,433 to $8,677 per QALY as the uptake rates increased.

Among all strategies, the questionnaire-based assessment combined with the FIT strategy reaches the lowest colonoscopy uptake threshold to be cost-effective under identical initial screening uptake. Setting the initial uptake rate as 10%, at least a 70% uptake rate of follow-up colonoscopy was needed to achieve cost-effectiveness at the WTP threshold of $12,246 per QALY gained. However, it is not cost-effective for the hypothetical non-invasive screening tests that meet the minimum criteria set by NMPA compared with no screening, even if all individuals with abnormal results adhered to the follow-up colonoscopy (*NMPA*_*min*_ ICER: $13,978 per QALY gained).

For non-invasive biomarker-based initial modalities, a higher colonoscopy uptake rate was critical to ensure the equivalent cost-effectiveness done by traditional strategies (cost-effectiveness noninferiority at 70% for *NMPA*_*min*_, 50% for blood-based tests, and 50% for mt-sDNA under the WTP threshold), irrespective of improvements in initial screening uptake. Furthermore, the adherence to colonoscopy also magnifies the benefits of improving the initial uptake rate. For example, the RF-FIT strategy achieved cost-effectiveness (ICER: $11,293/QALY) by maintaining a 70% colonoscopy uptake rate, requiring only a 10% initial screening rate. However, if colonoscopy uptake drops to 10%, initial screening uptake must exceed 90% to achieve an ICER of $15,934/QALY.

### Threshold analysis for cost and uptake rate

Threshold analysis indicates that reducing the cost or increasing the follow-up uptake for the non-invasive biomarker-based tests would improve overall cost-effectiveness (Figs F and G in S1 Text). The threshold cost of non-invasive biomarker-based tests for initial screening, e.g., mt-sDNA strategies, should decrease to $131.7 to be cost-effective compared to RF-FIT. Additionally, the follow-up colonoscopy uptake rate must be higher to be cost-effective to accommodate the non-invasive biomarker-based tests. With a 100% initial uptake rate, the *NMPA*_*min*_ strategy would require a follow-up colonoscopy rate of at least 80%−90% to meet the cost-effectiveness threshold, compared to 20%−30% for RF-FIT strategies (current reference value in China).

### Sensitivity analysis

**Fig 3:**
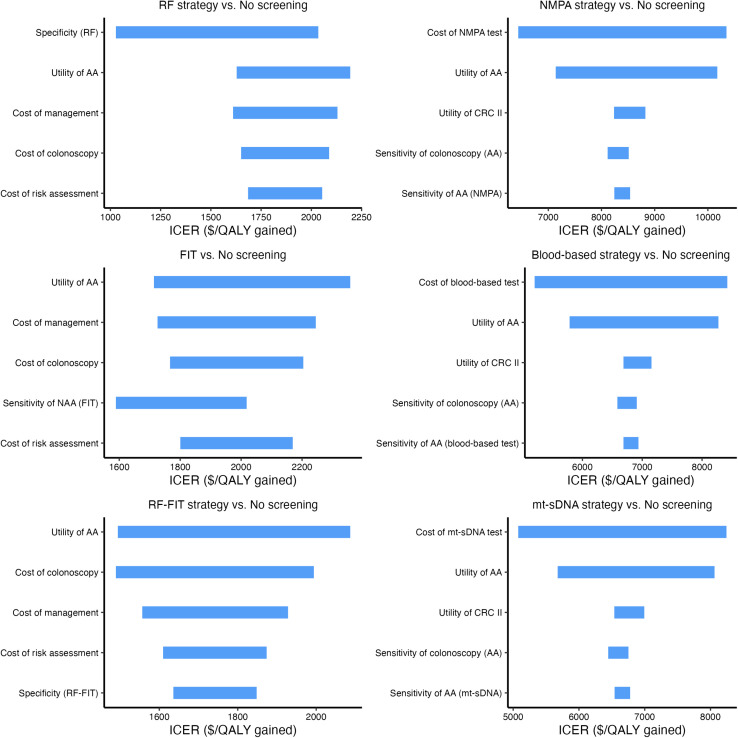
Deterministic one-way sensitivity analysis. Six different initial screening modalities were considered, including questionnaire-based risk factors assessment (RF), FIT, questionnaire-based risk factors assessment combined with FIT outcomes (RF-FIT), a hypothetical non-invasive test that meets the minimum criteria set by China National Medical Products Administration (*NMPA_min_*), blood-based strategies, and mt-sDNA test. The top five parameters with the greatest impact on the incremental cost-effectiveness ratio (ICER) are displayed. Costs are expressed in US dollars. The length of the blue bar represents the extent of ICER variation when the corresponding parameter changes. The cost-effectiveness threshold was set at $12,246 per QALY gained. Abbreviations: ICER, incremental cost-effectiveness ratio; QALYs, quality-adjusted life-years; NAA, non-advanced adenoma; AA, advanced Adenoma; CRC, colorectal cancer.

Extensive sensitivity analyses showed that the model was robust across a broad range of parameter values. Tornado diagrams showed the parameters with the greatest influence on the ICER (Fig 3). The model’s cost-effectiveness was most sensitive to the specificity and sensitivity of AA and NAA, health utility values of AA and CRC, and risk assessment specificity and cost.

## Discussions

Globally, quality improvement in adherence is increasingly recognized as essential for optimizing CRC programs. However, there remains no comprehensive analysis of multistep uptake impacts across the different screening stages based on China’s real-world data. Using a validated Markov model, we demonstrate that enhancing uptake at both initial risk assessment and colonoscopy stages yields significant cost-effectiveness. Furthermore, we quantified how enhanced uptake rates and colonoscopy follow-up translate into health and economic outcomes, providing evidence-based guidance for resource allocation and optimizing CRC screening programs.

Adherence is a key determinant of screening effectiveness and cost-effectiveness [55,56]. A population-based study showed that poor follow-up adherence diminished the mortality benefits of CRC screening [57]. A modeling study further demonstrated that increasing colonoscopy adherence after positive blood tests from 60% to 80% improved QALYs gains from 38–70 to 66–93 [56]. However, previous studies focused mainly on single-stage uptake, overlooking cumulative multistage adherence impacts [56,57]. We found that improving uptake rates across multiple stages, including high-risk identification and colonoscopy, yields nonlinear health gains that outpace incremental costs, thereby improving cost-effectiveness. This disproportionate benefit is driven in part by economies of scale in outreach, risk stratification, and system infrastructure. To support policy decisions, we developed a quantitative uptake-rate heatmap, identifying minimum uptake thresholds across multiple stages for cost-effective delivery, offering a practical tool for program design and quality monitoring.

The lack of head-to-head comparisons in pivotal studies limits direct assessment of competing strategies. Our evaluation of six primary screening approaches in China identified the questionnaire combined with the FIT strategy as the most cost-effective long-term option. This finding is consistent with population-based evidence that integrating risk assessment with FIT enhances colonoscopy uptake and overall screening efficiency [13,58,59]. While non-invasive biomarker approaches underperform conventional methods in cost-effectiveness, they remain better than no screening in cost-effectiveness, aligning with previous evaluations in diverse health settings [56,60,61]. These results indicate that biomarker-based screening potentially serves as a viable alternative for individuals unwilling to undergo conventional testing, potentially improving screening coverage. Diversifying screening options based on population preferences is, therefore, crucial for optimizing public health outcomes.

Non-invasive biomarker-based screening, while promising, continues to face challenges in efficiency, cost, and adherence. A modeling study in the US estimated that blood-based tests would require a 20-fold reduction in cost to be cost-effective compared to FIT [56]. In China, we identified a cost-effectiveness threshold of $131.7, slightly lower than the threshold in US modeling studies ($140 per-test) [61]. This disparity likely reflects differences in disease burden, cost structures, or WTP thresholds. Nevertheless, the threshold remains economically prohibitive in the Chinese context, where colonoscopy costs approximately $73, highlighting a significant barrier to widespread adoption, especially for individuals without health insurance. Future reductions in cost through economies of scale and localized manufacturing may improve the feasibility in resource-limited countries like China.

Failure to complete follow-up colonoscopy increases CRC mortality up to 3-fold [62], yet rates of follow-up colonoscopy for abnormal (non-colonoscopy) screening tests vary from 30% to 82% in screening trials. Our analysis showed that biomarker-based strategies require consistently higher follow-up colonoscopy rates (at least 50%) to achieve cost-effectiveness, compared to the RF-FIT strategy. This reflects the limited benefit of high-cost tests without follow-up colonoscopy completion. For current screening programs in China, prioritizing targeted adherence interventions over expensive non-invasive tests may represent a more efficient allocation of limited resources.

Achieving higher uptake rates will require considerable improvements in patient adherence and healthcare system capacity. Current uptake in China remains suboptimal due to resource disparities, awareness gaps, and logistical barriers, far below the U.S. National Colorectal Cancer Roundtable’s 80% target Europeans’ minimum acceptable threshold of 45% and recommends a threshold of at least 65% [63,64]. Closing this gap requires collaborative financial incentives among health systems and payers. Evidence suggests that outreach, navigation, education of patients or providers, reminders, and financial incentives can improve screening benefits [65]. Meta-analysis further validated navigation (RR = 2.01) and outreach (RR = 2.26) were the most effective interventions and could increase screening rates by approximately 20% [66]. Furthermore, expanding uptake metrics beyond single-round uptake to multiround participation and completion rates is crucial to ensure comprehensive program evaluation [67].

Currently, many countries recommend repeated CRC screening at regular intervals [3,11]. Our model primarily addressed the government-funded, one-time screening widely adopted in countries with limited screening resources and suboptimal follow-up adherence [68–70], which may underestimate the long-term effectiveness of repeated screening. Nevertheless, one-time screening reflects a pragmatic reality in many middle- and low-income countries, where broader coverage may yield greater population-level benefit than repeated screening of targeted individuals [71]. Four national cancer screening programs (NCSPs) are funded by the Chinese government (Table F in S1 Text), among which one-time screening is provided by most areas of the NCSPs [71]. Furthermore, evidence supporting the adoption of repeated screening remains limited in these settings. Evidence from some high-income countries also highlights suboptimal adherence to repeated screening: in the USA, only 38%–49% of participants with an initial negative result undergo repeat screening [72]. Therefore, optimizing strategies that ensure the delivery of at least one effective screening offer a practical complement to existing repeated screening paradigms.

An important strength is that our study comprehensively analyzes the impact of uptake rate in real-world scenarios across multistage (risk-assessment and follow-up colonoscopy) and a wide range of levels (10%–100%) for each screening strategy. We proposed a quantitative uptake rate heatmap to guide policy implementation. Referring to the threshold heatmap, policymakers can estimate the budgetary impact and incremental resource requirements for CRC implementation. Moreover, blood-based strategies have gradually become a promising technique for cancer screening for the advantage of lower hazards and higher compliance. Our study comprehensively evaluated the cost-effectiveness of non-invasive biomarker approaches, including mt-sDNA and blood-based test products approved by NMPA, providing valuable evidence to support the development, pricing, and implementation of new CRC screening methods.

Our study has several limitations. First, our model assumes all CRC cases arise from the adenoma-carcinoma sequence (given that the majority of CRC follow the adenoma-carcinoma pathway), for lack of natural history data of serrated polyps [73]. Besides, while we have endeavored to select high-quality epidemiological and screening parameters from China, population-based research on non-invasive screening methods is still relatively limited. Consequently, we conducted a systematic review of CRC screening products currently available in China to evaluate their effectiveness. As new technologies and revised guidelines emerge, it is essential to re-examine the definitions of current screening practices and update the analysis as necessary. Third, these thresholds are context-specific to China’s healthcare system and may not be directly generalizable to other settings due to differences in economic conditions, healthcare infrastructure. Caution should be exercised when interpreting these results across different health systems. Fourth, our model applied a life expectancy of 79 years, which may underestimate the long-term benefits of screening, as it does not capture individuals who survive beyond this age as a result of screening. Modeling a longer life span is constrained by the lack of detailed age-specific mortality data for those over 80; future updates could address this limitation as more robust data become available. Additionally, the one-time screening assumption, while limiting generalizability to settings with repeated screening programs, provides a realistic framework for areas with limited resources and suboptimal adherence [71]. Future model-based studies should also incorporate repeated screening evaluations as healthcare capacity expands and more evidence is available.

Our decision analysis demonstrated that multistage adherence is a key determinant of the long-term effectiveness and cost-efficiency of CRC screening. While questionnaire-based risk factors assessment combined with FIT is cost-effective under current conditions, optimal strategy selection should be guided by real-world adherence. Non-invasive screening methods may become viable with lower costs and higher follow-up compliance. This study provides strategic insight to strengthen the comprehensive management of CRC screening, contributing to global efforts toward CRC elimination, especially in countries with limited resources.

## Supporting information

S1 Text **Table A.** Nature history parameter of colorectal cancer. **Table B.** Cost of different screening strategies. **Table C**. Colorectal cancer screening guideline in China. **Table D.** Parameters review of multitarget stool DNA- or blood-based screening products approved by NMPA in China. **Table E.** CHEERS 2022 Checklist. **Table F**. Characteristics of the four national cancer screening programs in China. **Fig A.** Comparison between estimated and observed age-specific incidence and mortality of colorectal cancer. **Fig B.** Comparison between observed and model-calibrated stage distribution of colorectal cancer at diagnosis. **Fig C.** Cost-effectiveness plane for colorectal cancer screening strategies. **Fig D.** Cost-effectiveness acceptability curve of different screening strategies for colorectal cancer screening. **Fig E.** Impact of uptake rate on preventable colorectal cancer death of various colorectal cancer screening strategies. **Fig F.** Sensitivity analysis of the cost threshold of the biomarker-based screening test compared with the most cost-effective strategy. **Fig G.** Sensitivity analysis of the colonoscopy uptake rate threshold for the biomarker-based screening test compared with the most cost-effective strategy (RF-FIT strategy).
(DOCX)

## Abbreviations

CI: confidence interval
CRC: colorectal cancer
CRC-SIM: colorectal cancer screening simulation
FIT: fecal immunochemical test
ICER: incremental cost-effectiveness ratio
mt-sDNA: multitarget stool DNA
NCSPs: national cancer screening programs
*NMPA*_*min*_: minimum standards of China National Medical Products Administration
QALYs: quality-adjusted life years
RF: risk factors questionnaire
RR: relative risk
WTP: willingness-to-pay
