# Supplementary material for: The impact of adherence on colorectal cancer screening cost-effectiveness: A modeling study
Source: PLoS Med. 2025 Nov 26;22(11):e1004807. doi: 10.1371/journal.pmed.1004807 (PMC12654882; doi:10.1371/journal.pmed.1004807)
Supplement: S1 Text — Table A. Nature history parameter of colorectal cancer. Table B. Cost of different screening strategies. Table C. Colorectal cancer screening guideline in China. Table D. Parameters review of multitarget stool DNA- or blood-based screening products approved by NMPA in China. Table E. CHEERS 2022 Checklist. Table F. Characteristics of the four national cancer screening programs in China. Fig A. Comparison between estimated and observed age-specific incidence and mortality of colorectal cancer. Fig B. Comparison between observed and model-calibrated stage distribution of colorectal cancer at diagnosis. Fig C. Cost-effectiveness plane for colorectal cancer screening strategies. Fig D. Cost-effectiveness acceptability curve of different screening strategies for colorectal cancer screening. Fig E. Impact of uptake rate on preventable colorectal cancer death of various colorectal cancer screening strategies. Fig F. Sensitivity analysis of the cost threshold of the biomarker-based screening test compared with the most cost-effective strategy. Fig G. Sensitivity analysis of the colonoscopy uptake rate threshold for the biomarker-based screening test compared with the most cost-effective strategy (RF-FIT strategy). (DOCX) [file pmed.1004807.s001.docx]

S Text 1

Detailed Model Description of SIM-CRC

## **Model Overview and Assumptions**

A Markov cohort model, the Colorectal Cancer Screening Simulation (CRC-SIM) model, was developed in TreeAge Pro 2022 to simulate the natural history of colorectal cancer, screening, and treatment in a hypothetical population. The model includes 9 health states: normal epithelium, non-advanced adenoma (NAA), advanced adenoma (AA, referring to lesions with a size greater than or equal to 10 mm, containing villous components, or high-grade intraepithelial neoplasia), colorectal cancer (CRC) stages I-IV, CRC death, and other causes of death. In light of the clinical status of the Chinese population and large-scale cancer screening programs, the NAA and AA states are adopted to simulate the development of precancerous lesions in colorectal cancer.

At the start of the simulation, certain proportions of no neoplasm, NAA, AA, and CRC (stage I–IV) are assigned to the hypothetical population. All CRC develops through the adenoma-carcinoma sequence, accounting for more than 80% of CRC in Asia [1, 2]. Each time cycle of the model (one year), individuals have the probability of transitioning between health states as shown in **Fig A**. Only one transition is possible within each time cycle. Diagnosed CRC is set as a mutually exclusive health state in the model. If an individual were diagnosed, the disease stops progressing, and this individual should follow a disease pathway. Two absorbing states were modeled: dying from other causes and dying from CRC. Screening and removal of adenomas can prevent the occurrence of CRC, and the status of the person changes back to no lesion, while preclinical CRC will be regarded as screening-detected CRC. Subjects will then continue to have the probability to progress to the next states, as those without findings at screening. CRC-related death only occurs in individuals with diagnosed CRC. Individuals diagnosed with CRC who survive for more than 10 years will be regarded as survivors.

## **Model Parameters and Data Sources**

### **2.1 Natural History**

The transition probability between states was derived from a systematic review. Without screening, CRC patients may still be diagnosed through symptomatic presentation or incidental findings during the investigation of other health conditions. However, no published studies or datasets directly estimate these diagnosis probabilities. Therefore, we employed a model calibration approach to ascertain these parameter values [3]. The age-specified mortality (/100,000) of all-cause, colorectal cancer, and non-colorectal cancer in China was derived from the China Statistical Yearbook. Other causes of mortality were calculated as:

$Oth\_Cause_{Mort}\left( age \right) = All\_Cause_{Mort}\left( age \right)* (1 - (N\_CRC_{Deaths} (age) / N\_All_{Deaths}(age)))$

**Table A.** Nature history parameter of colorectal cancer

| Parameters | Base case value | Reference |
| --- | --- | --- |
| Normal to NAA | Age-dependent, range from 0.0006-0.0345 | Calibration [4] |
| NAA to AA | Age-dependent, range from 0.0147-0.0271 | Calibration [4, 5] |
| AA to preclinical CRC | Age-dependent, range from 0.0196-0.0519 | Calibration [4] |
| CRC I-II | 0.3017 | Calibration [6] |
| CRC II- III | 0.3492 |  |
| CRC III- IV | 0.2825 |  |
| Preclinical CRC I to diagnose | 0.0583 | Calibration [7] |
| Preclinical CRC II to diagnose | 0.2881 |  |
| Preclinical CRC III to diagnose | 0.6429 |  |
| Preclinical CRC IV to diagnose | 0.7500 |  |
| CRC I to died | 0.0226 | Calibration [8] |
| CRC II to died | 0.0383 |  |
| CRC III to died | 0.1375 |  |
| CRC IV to died | 0.1860 |  |

Abbreviations: advanced adenoma, AA; colorectal cancer, CRC; non-advanced adenoma, NAA.

### **2.2 Health Utility and Quality of Life Estimation**

Quality-adjusted life years (QALYs) were assessed as one of the primary outcomes. It was hypothesized that a healthy population devoid of adenomas would possess a health status QALY equivalent to 1. Colorectal-related lesions and treatments were assumed to be associated with reduced health status [9, 10]. Utility scores for each health state, ranging from 0 (death) to 1 (normal epithelium), were obtained from a hospital-based cross-sectional study on 300 newly diagnosed CRC Chinese patients using EQ-5D-5L (Table 2) [10, 11].

### **2.3 Costs**

From a societal perspective, cost components include (1) direct medical expenses from clinical diagnoses and treatments; (2) travel expenses; (3) productivity losses; and (4) costs specific to screening procedures [9]. Cost estimates were sourced from population-based colorectal cancer screening programs in China, as well as a multicenter, cross-sectional survey in 37 tertiary hospitals in 13 provinces across China (**Table B**) [9, 12]. All costs were adjusted based on the 2022 consumer price index in China and subsequently converted into US dollars (1 USD = 7 RMB). All unit costs were adjusted according to the consumer price index in China and then converted into US dollars.

### **2.4 Screening strategies and parameters**

In China, colorectal cancer screening usually follows a two-step principle, focusing first on high-risk groups and then on colonoscopy (**Table C**) [13-15]. In particular, we considered six strategies for high-risk population identification: (1) RF strategies: administering a CRC risk factors questionnaire; (2) FIT strategies: fecal immunochemical tests (FIT); (3) RF–FIT strategies: questionnaire-based risk factors assessment combined with FIT outcomes; (4) NMPA strategies: a hypothetical novel-based test that meets the minimum criteria set by China National Medical Products Administration (NMPA). (5) mt-sDNA strategies: stool-based tests such as the multitarget stool DNA (mt-sDNA) test. (6) Blood-based strategies: blood-based tests. Positive results were followed by colonoscopy tests. With colonoscopy, polyps are removed, and CRCs are biopsied if detected. The rationale for the strategy of screening only once was attributable to screening practices under limited financial support for CRC screening programs and low adherence. The summary parameter of screening strategies was listed in **Table D**.

**Table B.** **Cost of different screening strategies**

| **Parameters** | **Base case (US$)** | **95% CI (US$)** | **Reference** | |
| --- | --- | --- | --- | --- |
| **Screening cost** |  |  |  | |
| Publicity management | 10.70 | 8.03-13.38 | Estimated | |
| Risk assessment | 0.68 | 0.51-0.85 | [12] | |
| FIT | 2.17 | 1.63-2.71 |  |  |
| Colonoscopy | 72.47 | 54.35-90.59 |  |  |
| Travel cost from risk assessment | 0.07 | 0.07-0.09 | [9] | |
| Time lost from risk assessment | 2.22 | 0.32-4.12 |  |  |
| Pathology | 19.43 | 14.57-24.29 |  |  |
| Travel cost from clinical screening | 0.76 | 0.69-0.85 |  |  |
| Time lost from clinical screening | 7.60 | 2.85-12.35 |  |  |
| Treatment for complications | 154.57 | 115.93-193.21 |  |  |
| **Direct medical and non-medical cost of treatment** | | | |  |
| NAA | 189.29 | 141.97-236.61 | [9] | |
| AA |  |  |  |  |
| Surgery | 2657.3 | 2381.26-2933.34 |  |  |
| Endoscopic mucosal resection | 424.12 | 327.37-520.85 |  |  |
| Precent of AA receive surgery | 0.027 | 0.017-0.042 |  |  |
| CRC Stage I | 9944.76 | 9203.59-10685.94 |  |  |
| CRC Stage II | 10627.79 | 10099.34-11156.24 |  |  |
| CRC Stage III | 11928.97 | 11287.42-12570.51 |  |  |
| CRC Stage IV | 14665.51 | 13690.87-15640.15 |  |  |
| **Indirect cost of treatment** |  |  |  | |
| AA-surgery | 520.59 | 466.51-574.67 | [9] | |
| AA-endoscopic mucosal resection | 83.09 | 64.14-102.04 |  |  |
| CRC Stage I | 1895.56 | 1687.95-2103.17 |  |  |
| CRC Stage II | 1927.94 | 1775.58-2080.3 |  |  |
| CRC Stage III | 2441.06 | 2271.46-2610.67 |  |  |
| CRC Stage IV | 3158.44 | 2893.46-3423.41 |  |  |

Abbreviations: non-advanced adenoma, NAA; advanced Adenoma, AA; colorectal cancer, CRC; fecal immunochemical tests, FIT; confidence interval, CI.

### **2.5 Complications**

The use of colonoscopy can potentially result in complications, including major bleeding and perforation. The Endoscopic examination complication rate was derived from a previous study, with the base case value for the complication rate estimated at 0.00177, with a range of 0.00117 to 0.00239 [9].

**Table C**. Colorectal cancer screening guideline in China

| **Guideline** | **Year** | **Starting age** | **Stopping age** |
| --- | --- | --- | --- |
| CSO | 2023 | 40 years | 74 years |
| NCC | 2020 | 40 years (high risk) | 75 years |
| Colon Cancer society of CACA | 2018 | 40 years | 74 years |

Abbreviations: CACA, China Anti-Cancer Association; CSO, Chinese Society of Oncology; NCC, National Cancer Center of China;

**Table D.** Parameters review of multitarget stool DNA- or blood-based screening products approved by NMPA in China

| **Products** | **Targets** | **Sensitivity of CRC** | **Sensitivity of AA** | **Sensitivity of NAA** | **Specificity** | **Price** |
| --- | --- | --- | --- | --- | --- | --- |
| **multitarget stool DNA-based tests** | | | | | | |
| Product 1 [16] | KRAS & BMP3/NDRG4 | 91.90% | 63.50% | 15.80% | 87.10% | $285 |
| Product 2 [17] | SDC2 | 83.8% | 42.10% | 2.0%^#^ | 98.0% | $184 |
| Product 3 [18] | SDC2, ADHFE1, PPP2R5C | 87.10% | 46.2% | 11.10% | 90.10% | $128 |
| Product 4 [19] | SDC2 & TFPI2 | 95.31% | 63.16% | 33.64% | 88.39% | $82 |
| Product 5 ^*^ | miR-92a | 94.74% | 68.33% | 8.04%^#^ | 91.96% | $87 |
| Summary | | 91 % | 57% | 14% | 91% | $153 |
| **Blood-based tests** | |  |  |  |  |  |
| Product 6 [20] | Septin9, BCAT1, IKZF1, BCAN, VAV3 | 86.1% | 42.1% | 13.8% | 91.9% | - |
| Product 7 [21] | ctDNA | 86.6% | 79.0% | 11.9%^#^ | 88.1% | - |
| Summary | | 86% | 61% | 13% | 90% | $153  (Estimated) |

^#^ Sensitivity of NAA was calculated as (1-specificity), same as the assumption in Rosita’s model [22, 23].

* Real World Evidence and randomized controlled trials results published on the company website.

Abbreviations: non-advanced adenoma, NAA; advanced Adenoma, AA; colorectal cancer, CRC; China National Medical Products Administration, NMPA.

## **Model validation**

Our model has been validated for the Chinese population. Firstly, face validity was validated in our model. Specifically, the conception of our model structure follows the widely recognized adenoma–carcinoma sequence, consistent with prior validated models constructed by Germany [24] and China [3]. This structure better mirrors Chinese clinical practice and registry data, improving model realism and policy relevance.

We also conduct external validity by comparing model-derived cumulative incidence and modality, and cancer stage distribution to the national registry center in China. Our calibration process included the following steps:

(1) Calibration Range Based on Previous Literature: Initially, a wide range of starting transition rates was specified, manually drawing from previously published literature. This broad range was intended to ensure that the actual values would fall within the defined initial ranges for the calibration process.

(2) Selection of Calibration Targets: Multiple calibration targets were established to guide the calibration process. These included age-specific incidence and mortality rates for colorectal cancer, as derived from the Global Burden of Disease study. CRC Stage distribution from diagnosis colorectal cancer stage distribution in multicenter, hospital-based, cancer registration in China in 2016. All calibration targets were assumed to follow a normal distribution, with point estimates and 95% confidence intervals (CIs) obtained from the relevant literature [25].

(3) Parameter Search Strategies (Optimization): Bound optimization BY quadratic approximation (BOQA), a derivative-free optimization algorithm, was used to search for an optimal parameter solution by minimizing the weighted sum of squared differences between the observed and simulated data [26, 27]. This algorithm was designed to efficiently approximate optimal solutions without relying on gradient information. BOQA is particularly advantageous for complex or non-differentiable objective functions, offering strong numerical stability and fast convergence. The initial parameter set was manually adjusted to achieve an approximate fit to all independent data targets. This calibration was performed using a likelihood-based approach by BOQA in TreeAge software; In each simulation, one value for each age-specific incidence rate was randomly drawn from its initial range, creating a set of input values. The Markov model was then run with these inputs, and the resulting outputs were compared against the calibration targets. A goodness-of-fit score was defined as the sum of the log-likelihoods of the model outputs relative to the targets.

(4) Convergence Criteria and Stopping Rule: The calibration process was considered convergent when the convergence threshold of 5e-6 was achieved, indicating that the model had successfully fitted the data; **Fig A** shows the calibration outcome compared to the age-specific incidence and mortality rates, and **Fig B** shows the stage distribution of CRC cancer. The model parameters derived via the calibration process resulted in a good fit between model outcomes: Goodness of fit=4.97e-6.

**Fig A. Comparison between estimated and observed age-specific incidence and mortality of colorectal cancer.** The model was calibrated to reproduce these outcomes using a weighted least squares objective function.

**Fig B. Comparison between observed and model-calibrated stage distribution of colorectal cancer at diagnosis.** Abbreviations: colorectal cancer, CRC. The observed proportions of stage I–IV were derived from multicenter, hospital-based cancer registration data from the National Cancer Center of China. The model was calibrated to reproduce these proportions using a weighted least squares objective function. The calibrated model closely approximates the empirical distribution (Stage I: 15.22% vs. 15.20%; Stage II: 32.91% vs. 32.90%; Stage III: 33.53% vs. 33.50%; Stage IV: 18.34% vs. 18.30%).

**
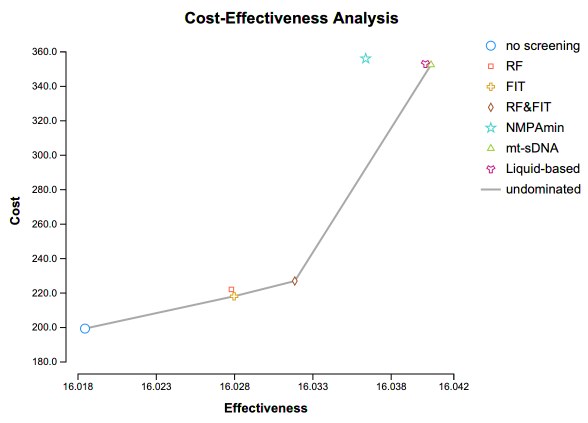
**

**Fig C. Cost-effectiveness plane for colorectal cancer screening strategies.** This figure illustrates the cost-effectiveness of alternative screening strategies compared to no screening, measured in terms of QALYs per person and the associated costs ($) per person. The strategies located on the frontier line are considered undominated. RF-FIT strategy is the most cost-effective strategy. The following preliminary screening strategies were considered, including questionnaire-based risk factors assessment (RF), fecal immunochemical tests (FIT), questionnaire-based risk factors assessment combined with FIT outcomes (RF-FIT), a hypothetical non-invasive test that meets the minimum criteria set by China National Medical Products Administration (NMPA_min_), blood-based strategies, and multitarget stool DNA (mt-sDNA) test. Abbreviations: colorectal cancer, CRC; Risk factor questionnaire, RF; fecal immunochemical test, FIT; multitarget stool DNA test, mt-sDNA.

**
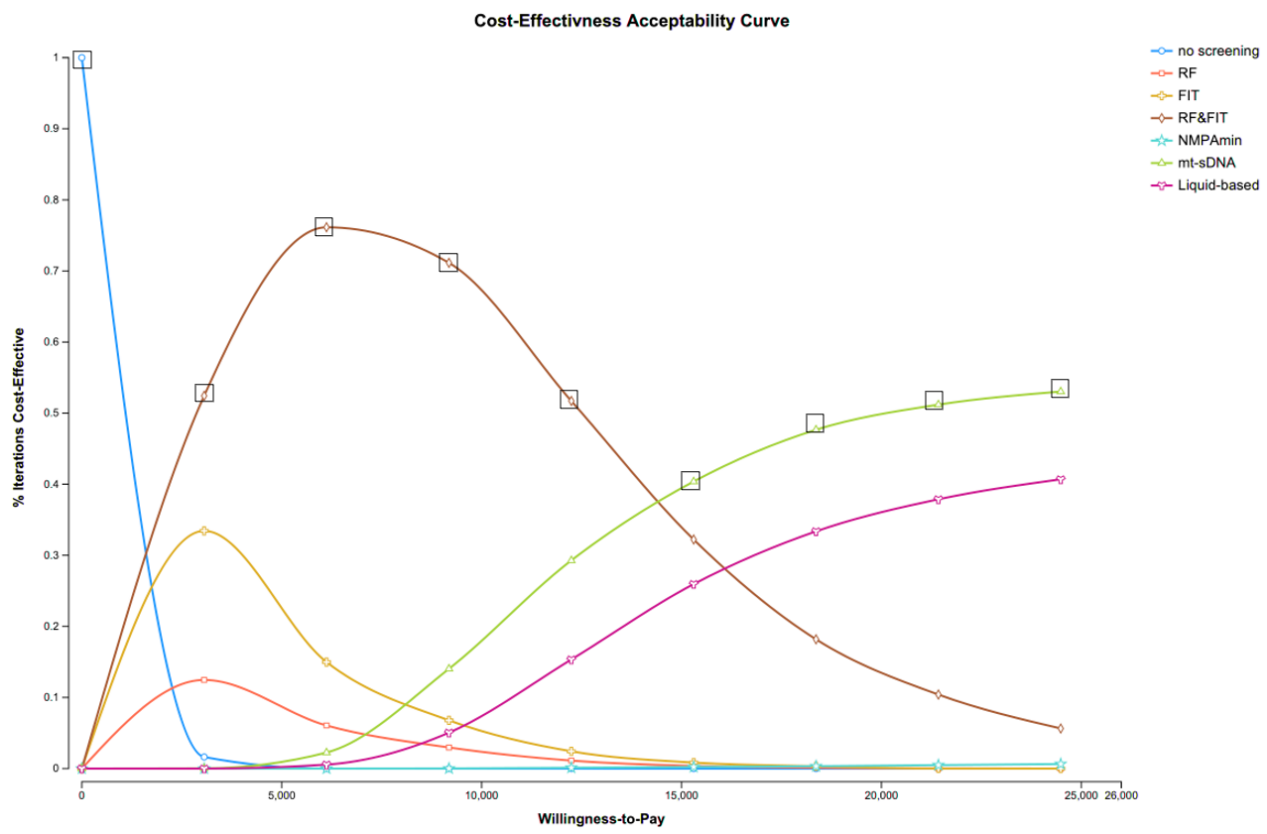
**

**Fig D**. **Cost-effectiveness acceptability curve of different screening strategies for colorectal cancer screening.** Abbreviations: colorectal cancer, CRC; Risk factor questionnaire, RF; fecal immunochemical test, FIT; multitarget stool DNA test, mt-sDNA.


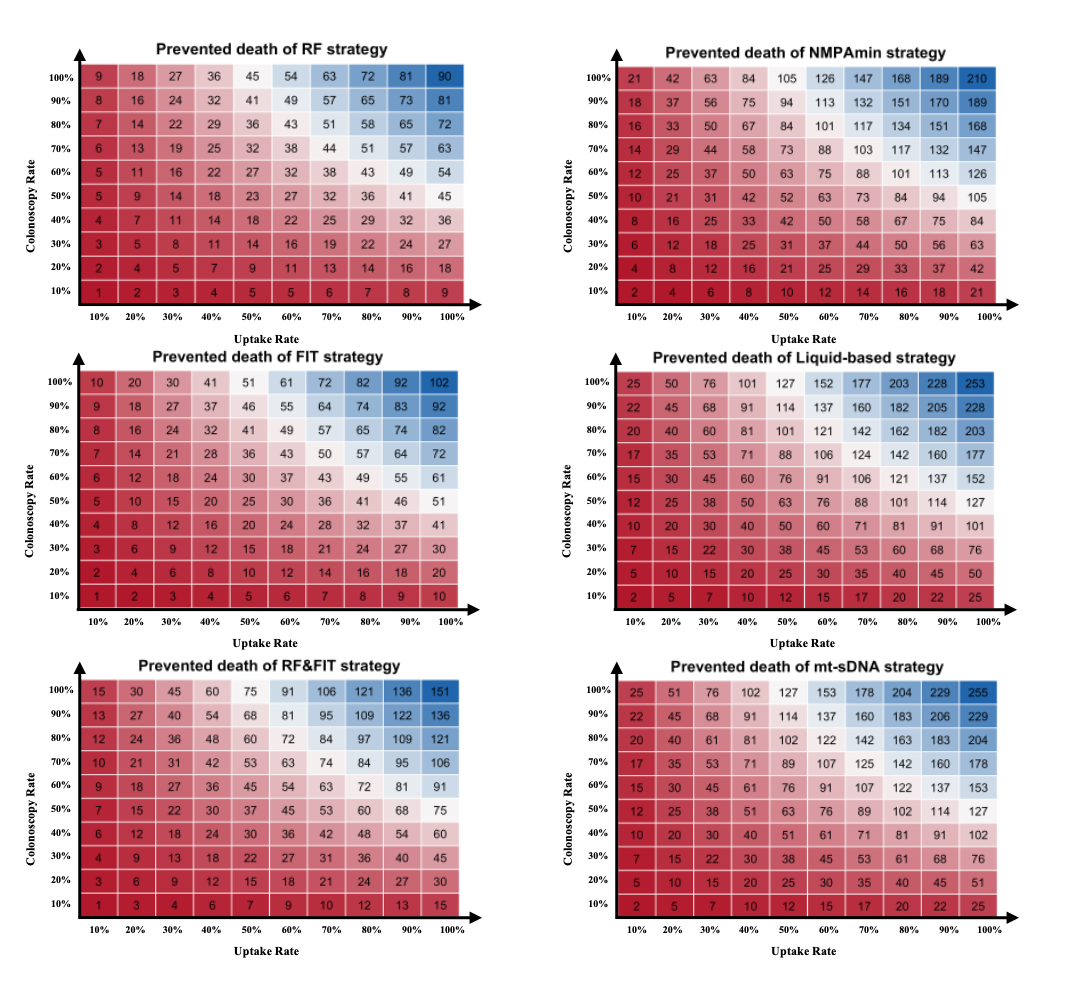


**Fig E.** **Impact of uptake rate on preventable colorectal cancer death of various colorectal cancer screening strategies.** The prevented death cases were calculated for each screening modality in comparison to no screening, taking into account the uptake rates for both initial screening and follow-up colonoscopy. The following initial screening strategies were considered, including questionnaire-based risk factors assessment (RF), fecal immunochemical tests (FIT), questionnaire-based risk factors assessment combined with FIT outcomes (RF-FIT), a hypothetical non-invasive test that meets the minimum criteria set by China National Medical Products Administration (NMPA_min_), blood-based strategies, and mt-sDNA test. Abbreviations: fecal immunochemical tests, FIT; China National Medical Products Administration, NMPA; multitarget stool DNA, mt-sDNA.


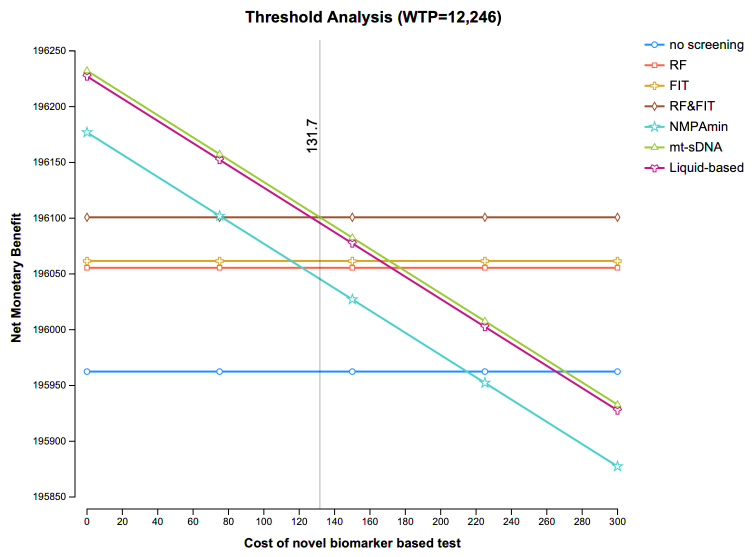


**Fig F. Sensitivity analysis of the cost threshold of biomarker-based screening test compared with the most cost-effective strategy.** The threshold unit costs of the biomarker test variants allowed for equal cost-effectiveness compared to FIT. The following preliminary screening strategies were considered, including questionnaire-based risk factors assessment (RF), fecal immunochemical tests (FIT), questionnaire-based risk factors assessment combined with FIT outcomes (RF-FIT), a hypothetical non-invasive test that meets the minimum criteria set by China National Medical Products Administration (NMPAmin), blood-based strategies, and multitarget stool DNA (mt-sDNA) test. Abbreviations: colorectal cancer, CRC; Risk factor questionnaire, RF; fecal immunochemical test, FIT; multitarget stool DNA test, mt-sDNA.


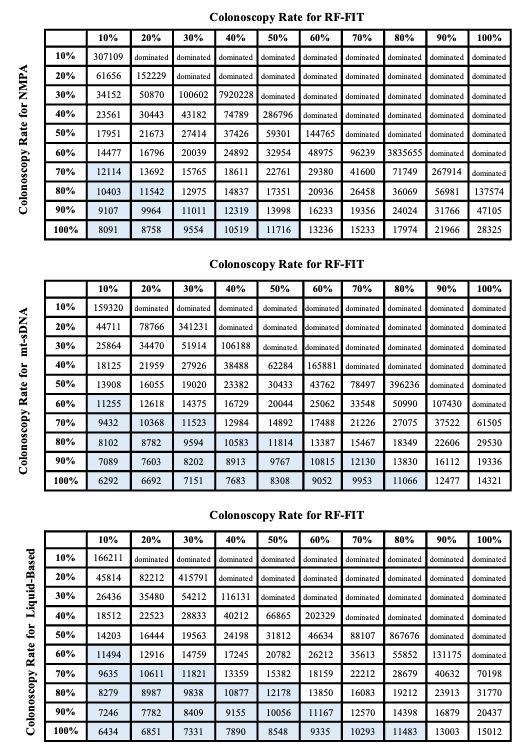


**Fig G. Sensitivity analysis of the colonoscopy uptake rate threshold for biomarker-based screening tests compared with the most cost-effective strategy (RF-FIT strategy)**. Abbreviations: colorectal cancer, CRC; Risk factor questionnaire, RF; fecal immunochemical test, FIT; multitarget stool DNA test, mt-sDNA; risk factors assessment combined with FIT, RF-FIT. Dominated refers to candidate strategies were dominated by risk factors assessment combined with FIT.

**Table E. CHEERS 2022 Checklist**

|  | Item | Guidance for Reporting | Reported in section |
| --- | --- | --- | --- |
| TITLE | | |  |
| Title | 1 | Identify the study as an economic evaluation and specify the interventions being compared. | Title Section |
| ABSTRACT | | |  |
| Abstract | 2 | Provide a structured summary that highlights context, key methods, results and alternative analyses. | Abstract Section |
| INTRODUCTION | | |  |
| Background and objectives | 3 | Give the context for the study, the study question and its practical relevance for decision making in policy or practice. | Section 1, paragraph 1-4 |
| METHODS | | |  |
| Health economic analysis plan | 4 | Indicate whether a health economic analysis plan was developed and where available. | NA |
| Study population | 5 | Describe characteristics of the study population (such as age range, demographics, socioeconomic, or clinical characteristics). | Section 2, paragraph 1 |
| Setting and location | 6 | Provide relevant contextual information that may influence findings. | Section 2, paragraph 1 |
| Comparators | 7 | Describe the interventions or strategies being compared and why chosen. | Section 2, paragraph 5 |
| Perspective | 8 | State the perspective(s) adopted by the study and why chosen. | Section 2, paragraph 3 |
| Time horizon | 9 | State the time horizon for the study and why appropriate. | Section 2, paragraph 1 |
| Discount rate | 10 | Report the discount rate(s) and reason chosen. | Section 2, paragraph 7 |
| Selection of outcomes | 11 | Describe what outcomes were used as the measure(s) of benefit(s) and harm(s). | Section 2, paragraph 7 |
| Measurement of outcomes | 12 | Describe how outcomes used to capture benefit(s) and harm(s) were measured. | Section 2, paragraph 7 |
| Valuation of outcomes | 13 | Describe the population and methods used to measure and value outcomes. | Section 2, paragraph 1 |
| Measurement and valuation of resources and costs | 14 | Describe how costs were valued. | Section 2, paragraph 3 |
| Currency, price date, and conversion | 15 | Report the dates of the estimated resource quantities and unit costs, plus the currency and year of conversion. | Section 2, paragraph 7 |
| Rationale and  description of model | 16 | If modelling is used, describe in detail and why used. Report if the model is publicly available and where it can be accessed. | Section 2, paragraph 1 |
| Analytics and assumptions | 17 | Describe any methods for analysing or statistically transforming data, any extrapolation methods, and approaches for validating any model used. | Section 2, paragraph 4 |
| Characterizing heterogeneity | 18 | Describe any methods used for estimating how the results of the study vary for sub-groups. | NA |
| Characterizing  distributional effects | 19 | Describe how impacts are distributed across different individuals or adjustments made to reflect priority populations. | NA |
| Characterizing uncertainty | 20 | Describe methods to characterize any sources of uncertainty in the analysis. | Section 3, paragraph 10 |
| Approach to engagement with patients and others affected by the study | 21 | Describe any approaches to engage patients or service recipients, the general public, communities, or stakeholders (e.g., clinicians or payers) in the design of the study. | NA |
| RESULTS | | |  |
| Study parameters | 22 | Report all analytic inputs (e.g., values, ranges, references) including uncertainty or distributional assumptions. | Section 2, paragraph 2 |
| Summary of main results | 23 | Report the mean values for the main categories of costs and outcomes of interest and summarise them in the most appropriate overall measure. | Section 3, paragraph 1 |
| Effect of uncertainty | 24 | Describe how uncertainty about analytic judgments, inputs, or projections affect findings. Report the effect of choice of discount rate and time horizon, if applicable. | Section 3, paragraph 7 |
| Effect of engagement with patients and others affected by the study | 25 | Report on any difference patient/service recipient, general public, community, or stakeholder involvement made to the approach or findings of the study | Section 4, paragraph 7 |
| DISCUSSION | | |  |
| Study findings, limitations, generalizability, and current knowledge | 26 | Report key findings, limitations, ethical or equity considerations not captured, and how these could impact patients, policy, or practice. | Section 4, paragraph 1,9 |
| OTHER RELEVANT INFORMATION | | | |
| Source of funding | 27 | Describe how the study was funded and any role of the funder in the identification, design, conduct, and reporting of the analysis | Finding Section |
| Conflicts of interest | 28 | Report authors conflicts of interest according to journal or  International Committee of Medical Journal Editors requirements. | Conflicts of interest Section |

From: Husereau D, Drummond M, Augustovski F, et al. Consolidated Health Economic Evaluation Reporting Standards 2022 (CHEERS 2022) Explanation and Elaboration: A Report of the ISPOR CHEERS II Good Practices Task Force. Value Health 2022;25. doi:10.1016/j.jval.2021.10.008

**Table F.** Characteristics of the four national cancer screening programme in China

| Program | Initiation year | Target cancer sites | Coverage | Primary screening  strategy |
| --- | --- | --- | --- | --- |
| Cancer Screening Programme in Rural Areas (pilot) | 2005 | 1. **Colorectum** 2. Esophagus 3. Stomach 4. Liver 5. Lung 6. Nasopharynx 7. Cervix | Organized screening in 249 counties or districts across 31 provinces and opportunistic screening in 748 hospitals across 31 provinces | Questionnaire assessment + FIT |
| Cancer Screening Programme in Huai River Areas | 2007 | 1. Esophagus 2. Stomach 3. Liver | 38 counties or districts in four provinces | / |
| Cervical Cancer and Breast Cancer Screening Programme for Women | 2009 | 1. Cervix 2. Breast | All counties or districts in 31 provinces | / |
| Cancer Screening Programme in Urban Areas (pilot) | 2012 | 1. **Colorectum** 2. Lung 3. Breast 4. Oesophagus 5. Stomach 6. Liver | 75 cities in 30 provinces | Questionnaire assessment + FIT |

**Reference**

1. Taherian M, Lotfollahzadeh S, Daneshpajouhnejad P, Arora K. Tubular Adenoma. StatPearls. Treasure Island (FL): StatPearls Publishing, StatPearls Publishing LLC.; 2025.

2. Makkar R, Pai RK, Burke CA. Sessile serrated polyps: cancer risk and appropriate surveillance. Cleve Clin J Med. 2012;79(12):865-71. doi: 10.3949/ccjm.79a.12034.

3. Chen Q, Fan Y, Huang K, Li W, Geldsetzer P, Bärnighausen T, et al. Cost-effectiveness of population-based screening for chronic obstructive pulmonary disease in China: a simulation modeling study. Lancet Reg Health West Pac. 2024;46. doi: 10.1016/j.lanwpc.2024.101065.

4. Li ZF, Huang HY, Shi JF, Guo CG, Zou SM, Liu CC, et al. A systematic review of worldwide natural history models of colorectal cancer: classification, transition rate and a recommendation for developing Chinese population-specific model. Zhonghua Liu Xing Bing Xue Za Zhi. 2017;38(2):253-60. doi: 10.3760/cma.j.issn.0254-6450.2017.02.024.

5. Zhou Q, Li HL, Li Y, Gu YT, Liang YR, Liu HZ, et al. Cost-effectiveness analysis of different screening strategies for colorectal cancer in Guangzhou, southern China: a Markov simulation analysis based on natural community screening results. BMJ Open. 2021;11(9):e049581. doi: 10.1136/bmjopen-2021-049581.

6. Wong CKH, Lam CLK, Wan YF, Fong DYT. Cost-effectiveness simulation and analysis of colorectal cancer screening in Hong Kong Chinese population: comparison amongst colonoscopy, guaiac and immunologic fecal occult blood testing. BMC Cancer. 2015;15(1):705. doi: 10.1186/s12885-015-1730-y.

7. Areia M, Mori Y, Correale L, Repici A, Bretthauer M, Sharma P, et al. Cost-effectiveness of artificial intelligence for screening colonoscopy: a modelling study. Lancet Digit Health. 2022;4(6):e436-e44. doi: 10.1016/S2589-7500(22)00042-5.

8. Wang R, Lian J, Wang X, Pang X, Xu B, Tang S, et al. Survival rate of colorectal cancer in China: A systematic review and meta-analysis. Front Oncol. 2023;13:1033154. doi: 10.3389/fonc.2023.1033154.

9. Xia C, Xu Y, Li H, He S, Chen W. Benefits and harms of polygenic risk scores in organised cancer screening programmes: a cost-effectiveness analysis. Lancet Reg Health West Pac. 2024;44:101012. doi: 10.1016/j.lanwpc.2024.101012.

10. Huang W, Yang J, Liu Y, Liu C, Zhang X, Fu W, et al. Assessing health-related quality of life of patients with colorectal cancer using EQ-5D-5L: a cross-sectional study in Heilongjiang of China. BMJ Open. 2018;8(12):e022711. doi: 10.1136/bmjopen-2018-022711.

11. Cheng CY, Calderazzo S, Schramm C, Schlander M. Modeling the Natural History and Screening Effects of Colorectal Cancer Using Both Adenoma and Serrated Neoplasia Pathways: The Development, Calibration, and Validation of a Discrete Event Simulation Model. MDM Policy Pract. 2023;8(1):23814683221145701. doi: 10.1177/23814683221145701.

12. Dong X, Du L, Luo Z, Xu Y, Wang C, Wang F, et al. Combining fecal immunochemical testing and questionnaire-based risk assessment in selecting participants for colonoscopy screening in the Chinese National Colorectal Cancer Screening Programs: A population-based cohort study. PLoS Med. 2024;21(2):e1004340. doi: 10.1371/journal.pmed.1004340.

13. Shaukat A, Kahi CJ, Burke CA, Rabeneck L, Sauer BG, Rex DK. ACG Clinical Guidelines: Colorectal Cancer Screening 2021. Am J Gastroenterol. 2021;116(3):458-79. doi: 10.14309/ajg.0000000000001122.

14. Chinese consensus on prevention of colorectal neoplasia (2021, Shanghai). J Dig Dis. 2022;23(2):58-90. doi: 10.1111/1751-2980.13079.

15. Schroy PC, 3rd, Wong JB, O'Brien MJ, Chen CA, Griffith JL. A Risk Prediction Index for Advanced Colorectal Neoplasia at Screening Colonoscopy. Am J Gastroenterol. 2015;110(7):1062-71. doi: 10.1038/ajg.2015.146.

16. Hu YT, Chen XF, Zhai CB, Yu XT, Liu G, Xiong ZG, et al. Clinical evaluation of a multitarget fecal immunochemical test-sDNA test for colorectal cancer screening in a high-risk population: a prospective, multicenter clinical study. MedComm. 2023;4(4):e345. doi: 10.1002/mco2.345.

17. Wang J, Liu S, Wang H, Zheng L, Zhou C, Li G, et al. Robust performance of a novel stool DNA test of methylated SDC2 for colorectal cancer detection: a multicenter clinical study. Clin Epigenetics. 2020;12(1):162. doi: 10.1186/s13148-020-00954-x.

18. Fang Y, Peng J, Li Z, Jiang R, Lin Y, Shi Y, et al. Identification of multi-omic biomarkers from Fecal DNA for improved Detection of Colorectal Cancer and precancerous lesions. MedRxiv. 2022:2022.11. 08.22282099.

19. Wang Z, Shang J, Zhang G, Kong L, Zhang F, Guo Y, et al. Evaluating the Clinical Performance of a Dual-Target Stool DNA Test for Colorectal Cancer Detection. J Mol Diagn. 2022;24(2):131-43. doi: 10.1016/j.jmoldx.2021.10.012.

20. Cai G, Cai M, Feng Z, Liu R, Liang L, Zhou P, et al. A Multilocus Blood-Based Assay Targeting Circulating Tumor DNA Methylation Enables Early Detection and Early Relapse Prediction of Colorectal Cancer. Gastroenterology. 2021;161(6):2053-6.e2. doi: 10.1053/j.gastro.2021.08.054.

21. Mo S, Dai W, Wang H, Lan X, Ma C, Su Z, et al. Early detection and prognosis prediction for colorectal cancer by circulating tumour DNA methylation haplotypes: A multicentre cohort study. EClinicalMedicine. 2023;55:101717. doi: 10.1016/j.eclinm.2022.101717.

22. Aziz Z, Wagner S, Agyekum A, Pumpalova YS, Prest M, Lim F, et al. Cost-Effectiveness of Liquid Biopsy for Colorectal Cancer Screening in Patients Who Are Unscreened. JAMA Netw Open. 2023;6(11):e2343392. doi: 10.1001/jamanetworkopen.2023.43392.

23. van den Puttelaar R, Nascimento de Lima P, Knudsen AB, Rutter CM, Kuntz KM, de Jonge L, et al. Effectiveness and Cost-Effectiveness of Colorectal Cancer Screening With a Blood Test That Meets the Centers for Medicare & Medicaid Services Coverage Decision. Gastroenterology. 2024;167(2):368-77. doi: 10.1053/j.gastro.2024.02.012.

24. Heisser T, Hoffmeister M, Brenner H. Effects of screening for colorectal cancer: Development, documentation and validation of a multistate Markov model. Int J Cancer. 2021;148(8):1973-81. doi: 10.1002/ijc.33437.

25. Chen Z, Chen J, Collins R, Guo Y, Peto R, Wu F, et al. China Kadoorie Biobank of 0.5 million people: survey methods, baseline characteristics and long-term follow-up. International journal of epidemiology. 2011;40(6):1652-66.

26. Kong CY, McMahon PM, Gazelle GS. Calibration of disease simulation model using an engineering approach. Value in Health. 2009;12(4):521-9.

27. Powell MJ. The BOBYQA algorithm for bound constrained optimization without derivatives. Cambridge NA Report NA2009/06, University of Cambridge, Cambridge. 2009;26:26-46.
